# The Associations between Asprosine, Clusterin, Zinc Alpha-2-Glycoprotein, Nuclear Factor Kappa B, and Peroxisome Proliferator-Activated Receptor Gamma in the Development of Complications in Type 2 Diabetes Mellitus

**DOI:** 10.3390/jcm13206126

**Published:** 2024-10-14

**Authors:** Abdulhalim Senyigit, Sinem Durmus, Omur Tabak, Aykut Oruc, Hafize Uzun, Iskender Ekinci

**Affiliations:** 1 Department of Internal Medicine, Faculty of Medicine, Istanbul Atlas University, 34403 Istanbul, Turkey; abdulhalim.senyigit@atlas.edu.tr; 2 Department of Medical Biochemistry, Faculty of Medicine, Katip Celebi University, 35620 Izmir, Turkey; durmus.sinem@gmail.com; 3 Internal Medicine Clinic, Kanuni Sultan Süleyman Training and Research Hospital, Health Sciences University, 34668 Istanbul, Turkey; omurtabak@yahoo.com.tr; 4 Department of Physiology, Cerrahpasa Faculty of Medicine, Istanbul University-Cerrahpasa, 34320 Istanbul, Turkey; aykut.oruc@icu.edu.tr; 5 Department of Biochemistry, Faculty of Medicine, Istanbul Atlas University, 34403 Istanbul, Turkey; 6 Department of Internal Medicine, Faculty of Medicine, Bezmialem Vakif University, 34093 Istanbul, Turkey; driskenderekinci@gmail.com

**Keywords:** T2DM, complications, asprosine, clusterin, zinc alpha-2-glycoprotein, nuclear factor kappa B, peroxisome proliferator-activated receptor gamma

## Abstract

**Objectives**: The aim of this study was to investigate the circulating levels of asprosin, clusterin, zinc-alpha-2-glycoprotein (ZAG), nuclear factor-kappa B (NF-κB), and peroxisome proliferator-activated receptor-gamma (PPAR-γ) in patients with T2DM in relation to microvascular and macrovascular complications. Measuring these biomarkers may provide insight into the pathophysiology of T2DM and indicate novel targets for the therapy of diabetes-related complications. **Methods**: A total of 260 subjects consisting of four groups: healthy controls (Group-1), T2DM patients without complications (Group-2), T2DM patients with microvascular complications (Group-3), and T2DM patients with macrovascular complications (Group-4). **Results**: The mean age of all subjects was 52.96 ± 6.4, 127 of whom were male. Asprosin, clusterin, and NF-κB levels were significantly higher, while ZAG and PPAR-γ levels were significantly lower in diabetic patients than healthy subjects (*p* < 0.01, for all). Asprosin (*p* < 0.01), clusterin (*p* < 0.01), and NF-κB (*p*: 0.002) levels were significantly higher and PPAR-γ (*p* < 0.01) level was significantly lower (*p* < 0.001) in Group-3 than Group-2. Asprosin (*p* < 0.01) and NF-κB (*p*: 0.011) levels were significantly higher while ZAG (*p* < 0.01) level was significantly lower in Group-4 than Group-2. Serum ZAG level was found lower in Group-4 than in Group-3 (*p* = 0.037). Further, the biomarkers presented significant correlation with biomarkers like HbA1c and HOMA-IR. It was observed that increasing serum asprosin, clusterin, and NF-κB levels and decreasing serum PPAR-γ levels were effective in the development of microvascular complications while the increased asprosin levels and decreased ZAG levels had a significant effect on the development of macrovascular complications in the binary logistic regression analysis. **Conclusions**: This study confirms that altered levels of asprosin, clusterin, ZAG, NF-κB, and PPAR-γ are associated with T2DM and its complications. These biomarkers reflect the pathophysiological processes of metabolic disturbance and inflammation in T2DM and, therefore, have the potential for use in targeted interventions to prevent and manage diabetes-related complications.

## 1. Introduction

Type 2 diabetes mellitus (T2DM) is a very common metabolic disorder characterized by the coexistence of two defects: pancreatic β-cell inability to secrete enough insulin and failure of insulin-sensitive tissues to compensate for this deficient insulin secretion properly [1]. Damage to the small blood vessels can lead to microvascular complications such as diabetic retinopathy, nephropathy, and neuropathy, while damage to the large vessels can lead to macrovascular complications like coronary artery disease, peripheral artery disease, and cerebrovascular disease. Pathologically, they are complex, including multiple factors such as oxidative stress, endothelial dysfunction, chronic inflammation, and metabolic abnormalities [2,3].

The goal of recent research has been to elucidate the mechanisms behind the vascular problems associated with T2DM. Asprosin is a recently identified fasting-induced glucogenic peptide that has been shown to implicate glucose homeostasis and may potentially link to insulin resistance (IR) and inflammation during vascular complication development [4]. In fact, patients with obesity, IR, and T2DM display pathologically elevated asprosin levels [5]. The multifunctional glycoprotein clusterin has been involved in lipid metabolism, apoptosis, and inflammation and, because of its roles in cellular stress response and tissue remodeling, also in the development of diabetes complications affecting both microvessels and macrovessels [6]. Plasma clusterin levels increase according to the severity of DM [7]. Zinc-alpha-2-glycoprotein (ZAG) also has a regulatory function in metabolic pathways and inflammatory responses. Therefore, ZAG, an adipokine involved in lipid metabolism and anti-inflammatory activities, has been proposed as a diagnostic tool for diabetic complications [8]. Serum ZAG levels are decreased in T2DM [9]. Nuclear factor-kappa B (NF-κB), an important transcription factor in immunologic and inflammatory responses, has also been suggested to be involved in the development of complications in diabetes [10,11]. Serum NF-κB-p65 levels are increased in T2DM [12]. It has been reported that peroxisome proliferator-activated receptor-gamma (PPAR-γ), acting as a nuclear receptor for controlling glucose and lipid metabolism, exerts anti-inflammatory and vasculoprotective effects, making it a potential therapeutic agent in preventing diabetic vascular complications [13,14,15].

Against the background described above, this study was undertaken to evaluate the association of circulating levels of asprosin, clusterin, ZAG, NF-κB, and PPAR-γ with complications in diabetic patients. Secondary aims are to provide information about the pathophysiology of diabetes and the development of possible new therapeutic strategies by examining the levels of molecules involved in some processes, such as lipid synthesis and degradation and IR.

## 2. Materials and Methods

### 2.1. Study Design

This multi-center prospective case–control study was conducted according to the guidelines of the Declaration of Helsinki. The study protocol was approved by the Bezmialem Vakif University Medical Faculty Clinical Research Ethics Committee (number of approval: E-54022451-050.04-162458, 26 August 2024). All subjects gave their informed consent for inclusion before they participated in the study. All subjects were of Turkish descent.

### 2.2. Subjects

This study was conducted with healthy subjects and patients with type 2 diabetes mellitus. The subjects included in the study were evaluated by dividing them into 4 groups, considering the presence of microvascular and macrovascular complications. Retinopathy, nephropathy, and neuropathy were considered microvascular complications, while macrovascular complications included cardiovascular, cerebrovascular, and peripheral vascular disease. The study groups are defined as follows:

Group-1: Healthy control group;

Group-2: Type 2 diabetes mellitus patients without complications;

Group-3: Type 2 diabetes mellitus patients with microvascular complications;

Group-4: Type 2 diabetes mellitus patients with macrovascular complications.

### 2.3. Exclusion Criteria

The patients having type 1 diabetes mellitus, diabetic ketoacidosis, any malignancy, severe liver disease, any active infection, acute renal failure, any chronic inflammatory disease, and chronic lung diseases (such as chronic obstructive pulmonary disease, asthma, bronchiectasis, pulmonary hypertension) as well as patients with malnutrition, organ transplant patients, patients on hemodialysis or peritoneal dialysis, and pregnant and breastfeeding women were excluded from study.

### 2.4. Data Collection

Age, gender, presence of diabetes in the family, and the duration of diagnosis of diabetes mellitus were recorded. Body weight was measured with a margin of error of 0.1 kg using an electronic scale (Seca digital scale, 0.1 precision, Hamburg, Germany), allowing only underwear to remain. Height was measured with a Harpenden stadiometer (Seca mod. 240 ce 0123, made in Hamburg, Germany) with an error margin of 0.1 cm. Height measurements were performed in the vertical position with bare feet, feet together and parallel, and shoulder and gluteal region in contact with the wall. Body mass index was calculated by the formula of weight in kilograms (kg) divided by height in meters (m) squared. Patients were categorized according to the BMI value. Measurements of systolic blood pressure (SBP) and diastolic blood pressure (DBP) were performed on the right arm by a mechanic sphygmomanometer after 15 min of resting. Mean arterial blood pressure (MAP) was calculated by the following formula: MAP = DBP + 1/3 (SBP − DBP). The patients’ medications were recorded. Retinopathy, nephropathy, neuropathy, cardiovascular disease, and cerebrovascular and peripheral vascular disease were noted, if present. In addition, the presence of dyslipidemia, hypertension, and metabolic syndrome was recorded. The data regarding the complications and comorbidities of the patients were obtained from the hospital automation system.

Microvascular and macrovascular complications were defined as follows:

Diabetic retinopathy: Diabetic retinopathy detected during eye examination by ophthalmoscopy, FFA, or stereoscopic digital and color film [16].

Diabetic neuropathy: The presence of symptoms or findings associated with diabetic neuropathy, such as peripheral neuropathy, gastroparesis, erectile dysfunction, resting tachycardia, and orthostatic hypotension or detected by electromyography during neurological examination [16].

Diabetic nephropathy: Persistent urine albumin-to-creatinine ratio values between 30 and 300 mg/g (moderately increased albuminuria) or persistent urine albumin-to-creatinine ratio values above 300 mg/g (severely increased albuminuria) [17].

Macrovascular complications: Atherosclerotic cardiovascular diseases such as coronary heart disease, myocardial infarction, heart failure, cerebrovascular disease, trans ischemic attack, peripheral artery disease, or diabetic foot ulcer that occurred after the diagnosis of diabetes were considered as macrovascular complications [18].

### 2.5. Sample Collection and Measurements

Fasting venous blood samples were drawn between 8 and 10 am after the subjects fasted overnight (10–12 h). Blood samples were drawn from the brachial veins in brachial fossa and placed into plain tubes and anticoagulant free tubes. The samples were centrifuged for 10 min at 4000 rpm at 4 °C. Biochemical tests were performed immediately. For the determination of other parameters, serum aliquots were frozen immediately and stored at −80 °C until required for further analysis.

### 2.6. Measurement of Serum Asprosin Levels

Serum asprosin levels were determined by using a commercially available human enzyme-linked immunosorbent assay (ELISA) kit (Sunred Bioscience, Cat. No: 201-12-7193, Shanghai, China) according to the manufacturer’s instructions. All samples were examined twice. Intra- and inter-assay coefficients of variation (%CVs) for asprosin were determined to be 10%.

### 2.7. Measurement of Serum Clusterin Levels

Serum clusterin levels were determined by using a commercially available human enzyme-linked immunosorbent assay (ELISA) kit according to the manufacturer’s instructions (Sunred Bioscience, Cat. No: 201-12-1190, Shanghai, China). All samples were examined twice. Intra- and inter-assay %CVs for clusterin were determined to be <10%.

### 2.8. Measurement of Serum Zinc Alpha-2-Glycoprotein (ZAG) Levels

Serum ZAG levels were determined by using a commercially available human enzyme-linked immunosorbent assay (ELISA) kit according to the manufacturer’s instructions (Biovendor Laboratorni Medicina, Cat. No: RD191093100R, Modrice, Czech Republic). All samples were examined twice. Intra- and inter-assay %CVs for ZAG were determined to be 4.0 and 6.5%, respectively.

### 2.9. Measurement of Serum Nuclear Factor Kappa B (NF-κB) Levels

Serum NF-κB levels were determined by using a commercially available human enzyme-linked immunosorbent assay (ELISA) kit according to the manufacturer’s instructions (MyBioSource, Cat. No: MBS724341, MyBioSource, Inc., San Diego, CA, USA). All samples were examined twice. Intra- and inter-assay %CVs for NF-κB were determined to be <9%.

### 2.10. Measurement of Serum Peroxisome Proliferator-Activated Receptor Gamma (PPAR-γ) Levels

Serum PPAR-γ levels were determined by using a commercially available human enzyme-linked immunosorbent assay (ELISA) kit according to the manufacturer’s instructions (MyBioSource, Cat. No: MBS263089, MyBioSource, Inc., San Diego, CA, USA). All samples were examined twice. Intra- and inter-assays %CVs for PPAR-γ were determined to be <10%.

Biochemical parameters such as ALT, AST, LDL, HDL, triglyceride, total cholesterol, total protein, albumin, uric acid, and creatinine were determined using enzymatic methods (Architect i2000, Abbott Park, IL, USA). The vitamin D levels were measured by enzyme-linked fluorescent assay on the Mini Vidas (Biomerieux, Paris, France). Insulin levels were measured by the electrochemiluminescence immunoassay (ECLIA) method on Roche-Hitachi E170 (Roche/Hitachi MODULAR Analytics Combination Systems, Roche Diagnostics, Indianapolis, IN, USA). HbA1c determination was based on HPLC (Variant Turbo II, Bio-Rad Laboratories, Inc., Hercules, CA, USA). Homeostasis model assessment for insulin resistance (HOMA-IR) was calculated by using the following formula:

HOMA-IR = Fasting glucose (mg/dL) × Fasting insulin (mU/L)/405

Evaluation of diabetic nephropathy was performed by calculating glomerular filtration rate (GFR). GFR was calculated by the Cocroft-Gault (CG) formula using gender and body surface area [19].

GFR (MDRD) = 175 × standardized S_cr_ ^−1.154^ × age^−0.203^ × 1.212 [if black] × 0.742 [if female]

Microalbuminuria was determined from three separate 24 h urine samples collected at least one month apart by the nephelometric method. Excretion of 30–300 mg/day albumin in urine in at least two of three samples was considered microalbuminuria [20].

### 2.11. Statistical Analysis

SPSS version 22.0 for Windows (IBM Corporation, Chicago, IL, USA) was used for statistical analyses. Categorical variables were presented as numbers and percentages, and numerical variables were presented as mean ± standard deviation. The Shapiro-Wilk test was used to determine how a variable was distributed. The chi-square test was used to compare categorical data between groups. When a numerical variable was compared between more than two groups, the ANOVA test was used for parameters showing normal distribution, and the Kruskal–Wallis test was used for parameters showing abnormal distribution. Pearson correlation analysis was used for variables showing normal distribution, and Spearman correlation analysis was used for variables showing abnormal distribution. Binary logistic regression analysis was performed to determine whether parameters such as asprosin, clusterin, ZAG, NF-κB, and PPAR-γ have any effects on the occurrence of microvascular and macrovascular complications. A *p* value < 0.05 was considered significant.

## 3. Results

This study was conducted with 260 subjects, 127 male and 133 female. Age, gender distribution, family history of diabetes, age at diabetes diagnosis, and anthropometric measurements between the groups are presented in Table 1 below. Laboratory test result analyses between the groups are shown in Table 2.

Diabetic patients were distributed according to body mass index as follows: normal weight (n = 3), overweight (n = 115), class 1 obesity (n = 75), class 2 obesity (n = 6), and class 3 obesity (n = 1). Of the patients, 93 had hypertension, 97 had dyslipidemia, and 190 had metabolic syndrome.

The drugs used and the frequency of use were as follows: insulin therapy (n = 119), metformin (n = 177), dipeptidyl peptidase-4 inhibitor (n = 92), pioglitazone (n = 1), sulfonylurea (n = 68), statins (n = 97), fenofibrate (n = 13), gabapentin (n = 9), angiotensin converting enzyme inhibitors/angiotensin receptor blockers (n = 66), calcium channel blockers (n = 45), beta blockers (n = 47), diuretics (n = 38), and alpha blockers (n = 13).

The levels of asprosin, clusterin, ZAG, NF-κB, and PPAR-γ were compared across the four groups: healthy controls (Group-1), type 2 diabetes mellitus (T2DM) patients without complications (Group-2), T2DM patients with microvascular complications (Group-3), and T2DM patients with macrovascular complications (Group-4).

Asprosin levels were significantly higher in Group-2, Group-3, and Group-4 compared to Group-1 (*p* < 0.001). Clusterin levels followed a similar trend, with significantly higher levels in Group-2, Group-3, and Group-4 compared to Group-1 (*p* < 0.001). Conversely, ZAG levels were significantly lower in Group-2, Group-3, and Group-4 than in the control group (*p* < 0.001). NF-κB levels were also significantly elevated in Group-2, Group-3, and Group-4 compared to Group-1 (*p* < 0.001). In contrast, PPAR-γ levels were significantly reduced in Group-2, Group-3, and Group-4 compared to the control group (*p* < 0.001) (Table 3).

Pairwise comparisons showed significant differences between Group-1 and the other groups for all parameters (*p* < 0.001). Group-2 and Group-3 showed significant differences in asprosin, clusterin, NF-κB, and PPAR-γ levels. Similarly, Group-2 and Group-4 differed significantly in asprosin, ZAG, and NF-κB levels. However, comparisons between Group-3 and Group-4 revealed significant differences only for ZAG levels (*p* = 0.037) (Table 3).

Correlation analysis revealed that asprosin levels were positively correlated with clusterin (r = 0.287, *p* < 0.001), HbA1c (r = 0.292, *p* < 0.001), and HOMA-IR (r = 0.212, *p* = 0.003). Clusterin also showed a positive correlation with HbA1c (r = 0.300, *p* < 0.001) and HOMA-IR (r = 0.287, *p* < 0.001) but was inversely correlated with PPAR-γ (r = −0.412, *p* < 0.001). ZAG levels were inversely correlated with HbA1c (r = −0.197, *p* = 0.005) and HOMA-IR (r = −0.295, *p* < 0.001). NF-κB did not show significant correlations with HbA1c or other biomarkers. PPAR-γ was inversely correlated with microalbuminuria (r = 0.362, *p* < 0.001). These findings suggest complex interactions between these biomarkers and T2DM metabolic parameters (Table 4).

In the binary logistic regression, it was observed that increasing serum asprosin, clusterin, and NF-κB levels and decreasing serum PPAR-γ levels were statistically significantly effective in the development of microvascular complications, while the increased serum asprosin and ZAP levels had a significant effect on the development of macrovascular complications (Table 5).

## 4. Discussion

This study focused on circulating levels of asprosin, clusterin, ZAG, NF-κB, and PPAR-γ in diabetic patients, evidencing remarkable changes in such biomarkers, with special emphasis on the relationships with complications related to T2DM. Results from our current study showed that asprosin levels are considerably higher in diabetic subjects, especially in complicated patients, when compared to healthy subjects. This is in agreement with previous studies indicating a positive relationship of serum asprosin with metabolic parameters, including triglycerides and insulin resistance [21,22,23]. The high level of asprosin in T2DM patients indicates that it may be used as a biomarker for glucose dysregulation and insulin resistance, both key components of the pathophysiology of diabetes. This is reflected in a number of studies [24,25]. In the current study, specifically, asprosin and clusterin levels were markedly elevated in T2DM patients, regardless of the presence of complications, compared to healthy controls. This finding aligns with previous research indicating that asprosin, an adipokine implicated in glucose metabolism, is often elevated in states of insulin resistance and metabolic dysfunction [26]. The increased levels of asprosin in Groups 2, 3, and 4 suggest a potential role in the pathophysiology of T2DM and its complications, as elevated asprosin has been associated with increased insulin resistance and impaired glucose homeostasis [27,28].

Clusterin is another biomarker researched in the current study, and its level was significantly lower in healthy controls than in diabetic patients. This also agrees with the literature; clusterin may exert a role in oxidative stress and inflammation, features of diabetic conditions [7,29,30]. An increase in clusterin levels among diabetic patients may show the impairment of the protective system against complications of diabetes, such as diabetic nephropathy and cardiovascular diseases [30,31]. Furthermore, the association between clusterin levels and diabetic complications may explain the mechanisms underlying diabetes-related pathologies.

In the current study, conversely, ZAG levels were significantly lower in all T2DM groups compared to healthy controls. This observation is consistent with the literature indicating that ZAG, a protein involved in lipid metabolism and insulin sensitivity, tends to decrease in states of obesity and insulin resistance [32]. A decline in ZAG levels in T2DM patients can reflect changes in metabolic processes featuring characteristic fat mobilization impairment and increased adiposity due to insulin resistance [6,33]. Moreover, the significant reduction of ZAG in T2DM patients may contribute to the dysregulation of lipid metabolism, further exacerbating the complications associated with diabetes [33]. Elsheikh et al. [34] reported a strong positive correlation between urinary ZAG concentrations and urinary albumin–creatinine ratio. They also suggested that urinary ZAG may be a useful biomarker for the early diagnosis of diabetic nephropathy in patients with T2DM, as urinary ZAG appears earlier than albuminuria. Therefore, ZAG, with its association with metabolic dysregulation, carries tremendous potential as a target molecule in the management of diabetes and its complications.

In the current study, compared with healthy controls, the concentration of NF-κB, representing the pro-inflammatory marker, was significantly higher in diabetic patients. This observation is of high relevance, as NF-κB mediates inflammatory responses and has been implicated in the pathogenesis of insulin resistance and T2DM [12,23,24,35]. More importantly, NF-κB was highly upregulated in the current study and is considered one of the major regulators of inflammatory processes implicated in the pathogenesis of diabetic complications; hence, the persistent activation of NF-κB in T2DM may contribute to the inflammatory milieu that characterizes both microvascular and macrovascular complications, reinforcing the need for therapeutic strategies targeting this pathway [36].

In diabetic patients, the levels of PPAR-γ were also much lower compared to healthy controls. This is in line with the hypothesis that PPAR-γ has an important role in glucose and lipid metabolism [37,38]. The reduction of PPAR-γ in T2DM patients may contribute to dysregulation in insulin sensitivity and promote inflammatory processes by virtue of its role, as reported to inhibit NF-κB activities [37]. Thus, this relationship has been identified for the potential therapeutic use of PPAR-γ agonists, which could improve insulin sensitivity and reduce inflammation in diabetic patients [36,37,38,39].

In the current study, pairwise comparisons further elucidated the relationships between these biomarkers, particularly highlighting significant differences in asprosin, clusterin, NF-κB, and PPAR-γ levels between Groups 2 and 3, as well as between Groups 2 and 4. These findings suggest that the presence of microvascular and macrovascular complications may further exacerbate the dysregulation of these biomarkers, indicating a potential gradient of severity in metabolic dysfunction among T2DM patients [38]. Notably, the significant difference in ZAG levels between Groups 3 and 4 underscores the potential role of ZAG in differentiating between the types of complications experienced by T2DM patients.

### Limitaion of Study

Since there are limited data in the literature on these parameters, which have only recently been used, we believe that the sample size of our study is large enough to be able to comment on these parameters. Because a large portion of our study group was overweight or obese, the findings we obtained are insufficient when evaluating non-obese diabetic patients. Considering that almost all of the patients we included in the study (including the group that had not yet developed complications) had metabolic syndrome, it is thought that these parameters may also be related to dyslipidemia, hypertension, and obesity in addition to their relationship with DM complications. New studies can be organized in non-obese diabetic patients without metabolic syndrome to clarify the confusion on this issue. Another shortcoming of our study is that the patients in our study group were using many medications, and the possible effects of these drug groups on the parameters in our study are not yet known. ZAG levels are not evaluated in urine.

## 5. Conclusions

The alterations in asprosin, clusterin, ZAG, NF-κB, and PPAR-γ levels across the studied groups highlight the complex interplay of metabolic dysregulation and inflammation in T2DM. These biomarkers not only reflect the underlying pathophysiology of diabetes but also present potential avenues for future research aimed at developing targeted interventions to mitigate the complications associated with this chronic disease. It was observed that increasing serum asprosin, clusterin, and NF-κB levels and decreasing serum PPAR-γ levels were effective in the development of microvascular complications, while the increased serum asprosin and decreased ZAG levels had a significant effect on the development of macrovascular complications in the binary logistic regression analysis.

## Figures and Tables

**Table 1:** Demographic data of the groups.

Parameter	Group-1 n = 60	Group-2 n = 60	Group-3 n = 70	Group-4 n = 70	*p*
Age (year)	51.73 ± 6.25	53.23 ± 6	53.4 ± 5.84	53.37 ± 7.32	0.405
Male gender n (%)	29 (48.3)	39 (65)	28 (40)	31 (44.3)	0.029
Presence of diabetes in family n (%)	-	50 (83.3)	54 (77.1)	60 (85.7)	<0.001
Duration of DM (year)	-	9.01 ± 5.64	9.42 ± 6.65	7.68 ± 5.53	<0.001
Weight (kg)	70.03 ± 9.38	82.28 ± 7.92	79.32 ± 9.21	78.82 ± 11.87	<0.001
BMI (kg/m^2^)	23.96 ± 1.38	29.86 ± 2.83	29.19 ± 2.31	28.56 ± 4.51	<0.001
SBP (mmHg)	117.65 ± 8.67	138.15 ± 19.02	137.6 ± 16.09	138.37 ± 18.28	<0.001
DBP (mmHg)	75.21 ± 6.51	79.36 ± 8.33	80.05 ± 9.13	79.97 ± 8.97	0.003
MAP (mmHg)	89.36 ± 6.71	98.96 ± 10.19	99.23 ± 10.22	99.43 ± 10.29	<0.001

Abbreviations: DM: Diabetes mellitus. BMI: Body mass index. SBP: Systolic blood pressure. DBP: Diastolic blood pressure. MAP: Mean arterial pressure.

**Table 2:** Analysis of laboratory parameters between groups.

Parameter	Group-1 n = 60	Group-2 n = 60	Group-3 n = 70	Group-4 n = 70	*p*
Glucose (mg/dL)	88.12 ± 6.65	147.18 ± 41.45	154.92 ± 70.11	174 ± 67.79	<0.001
HOMA-IR	1.77 ± 0.53	5.9 ± 4.77	7.10 ± 12.15	5.94 ± 4.73	<0.001
Creatinine (mg/dL)	0.74 ± 0.16	0.87 ± 0.91	0.97 ± 0.90	0.76 ± 0.21	0.139
GFR (ml/dk/1.73 m^2^)	126.38 ± 26.27	100.06 ± 10.03	82.61 ± 15.76	124.91 ± 36.13	<0.001
Microalbuminuria (mg/L)	1.13 ± 0.1.06	1.77 ± 2.18	30.95 ± 46.08	1.16 ± 1.37	<0.001
ALT (U/L)	25.54 ± 13.81	23.78 ± 15.73	23.41 ± 15.51	24.37 ± 13.4	0.586
AST (U/L)	19.95 ± 5.75	18.18 ± 7.26	18.83 ± 7.34	22.38 ± 12.12	0.246
LDL cholesterol (mg/dL)	101.79 ± 16.28	110.21 ± 36.94	131.56 ± 38.93	125.12 ± 37.13	<0.001
HDL cholesterol (mg/dL)	53.54 ± 13.37	47.88 ± 13.63	43.48 ± 10.89	43.31 ± 8.98	<0.001
Triglyceride (mg/dL)	84.05 ± 27.61	125.75 ± 39.65	143.80 ± 70.21	183.91 ± 114.73	<0.001
Total cholesterol (mg/dL)	169.85 ± 16.01	198.66 ± 23.41	216.27 ± 35.92	214.82 ± 39.21	<0.001
HbA1c (%)	5.61 ± 0.32	7.89 ± 1.5	8.59 ± 1.58	8.84 ± 1.93	<0.001
Insulin (mIU/L)	8.14 ± 2.36	14.68 ± 7.82	16.27 ± 13.28	12.81 ± 6.41	<0.001
25-OH Vitamin D (µg/L)	30.02 ± 3.99	19.0 ± 13.7	19.79 ± 13.86	19.21 ± 14.48	<0.001
Total protein (g/L)	7.34 ± 0.31	7.16 ± 0.34	6.82 ± 0.55	7.19 ± 0.57	<0.001
Albumin (g/L)	4.51 ± 0.28	4.48 ± 0.26	4.10 ± 0.51	4.54 ± 0.35	<0.001
Uric acid (mg/dL)	4.87 ± 1.61	5.14 ± 1.47	5.4 ± 1.4	4.96 ± 1.47	0.181
Sodium (mmol/L)	134.08 ± 13.87	139.93 ± 2.52	138.71 ± 3.64	139.81 ± 2.38	0.219
Potassium (mmol/L)	5.22 ± 1.97	4.61 ± 0.42	4.52 ± 0.57	4.56 ± 0.39	0.775
Calcium (mg/dL)	9.43 ± 0.43	9.50 ± 0.44	9.26 ± 0.51	9.30 ± 0.43	0.01
Phosphorus (mg/dL)	3.23 ± 0.46	4.06 ± 4.61	3.51 ± 0.60	3.41 ± 0.5	0.112
Hemoglobin (g/dL)	13.85 ± 1.57	13.39 ± 1.37	13.54 ± 1.72	13.91 ± 1.99	0.263
Platelet count (10^3^/uL)	313.25 ± 63.21	274.16 ± 63.82	277.42 ± 54.55	278.60 ± 77.78	0.006
CRP (mg/dL)	1.42 ± 1.38	2.91 ± 2.11	13.90 ± 21.86	5.06 ± 6.64	<0.001

Abbreviations: HOMA: Homeostasis Model Assessment. GFR: Glomerular Filtration Rate. ALT: Alanine Transaminase. AST: Aspartate Transaminase. LDL: Low-Density Lipoprotein. HDL: High-Density Lipoprotein. CRP: C-Reactive Protein.

**Table 3:** Serum Asprosin, Clusterin, ZAG, NF-κB, and PPAR-γ levels in subgroups.

Parameter	Group-1 n = 60	Group-2 n = 60	Group-3 n = 70	Group-4 n = 70	*p*
Asprosin (ng/mL)	13.59 ± 3.01	30.39 ± 8.29	44.83 ± 8.95	41.47 ± 10.08	<0.001
Clusterin (ng/mL)	37.66 ± 6.95	44.58 ± 7.67	50.21 ± 7.03	48.75 ± 0.08	<0.001
ZAG (ng/mL)	60.23 ± 11.63	42.75 ± 8.26	39.69 ± 8.17	36 ± 7.54	<0.001
NF-κB (ng/mL)	1.29 ± 0.69	2.11 ± 0.9	2.61 ± 0.74	2.54 ± 0.74	<0.001
PPAR-γ (ng/mL)	33.18 ± 4.91	24.54 ± 3.76	20.79 ± 4.83	22.47 ± 5.79	<0.001
Differences between groups					
*p* values for	Asprosin	Clusterin	ZAG	NF-κB	PPAR-γ
Group-1 vs. Group-2	<0.001	<0.001	<0.001	<0.001	<0.001
Group-1 vs. Group-3	<0.001	<0.001	<0.001	<0.001	<0.001
Group-1 vs. Group-4	<0.001	<0.001	<0.001	<0.001	<0.001
Group-2 vs. Group-3	<0.001	<0.001	0.2	0.002	<0.001
Group-2 vs. Group-4	<0.001	0.09	<0.001	0.011	0.08
Group-3 vs. Group-4	0.74	0.655	0.037	0.94	0.182

Abbreviations: NF-κB: nuclear factor kappa B; PPAR-γ: peroxisome proliferator-activated receptor gamma; ZAG: zinc alpha-2-glycoprotein.

**Table 4:** Correlation analysis in patient groups.

Parameter		Asprosin	Clusterin	ZAG	NF-κB	PPAR-γ
Clusterin	r	0.287	0.287	−0.049	0.033	−0.412
	*p*	<0.001	<0.001	0.493	0.640	<0.001
ZAG	r	−0.069	−0.049		−0.130	0.017
	*p*	0.329	0.493		0.067	0.812
NF-κB	r	0.033	0.072	−0.130		−0.109
	*p*	0.640	0.314	0.067		0.124
PPAR-γ	r	−0.229	−0.412	0.017	−0.109	
	*p*	0.001	<0.001	0.812	0.124	
Age	r	0.004	−0.023	0.088	0.015	−0.098
	*p*	0.958	0.747	0.214	0.832	0.167
BMI	r	−0.003	−0.022	−0.085	−0.017	−0.048
	*p*	0.966	0.756	0.233	0.815	0.501
SBP	r	−0.114	−0.51	−0.22	−0.006	−0.009
	*p*	0.109	0.471	0.758	0.938	0.899
DBP	r	−0.078	−0.93	−0.66	0.057	−0.054
	*p*	0.270	0.192	0.355	0.424	0.451
LDL Cholesterol	r	0.175	0.019	−0.084	0.043	0.033
	*p*	0.013	0.79	0.237	0.548	0.645
HDL Cholesterol	r	−0.180	−0.085	0.038	0.090	0.054
	*p*	0.011	0.232	0.591	0.208	0.444
Triglyceride	r	0.104	0.028	−0.018	−0.031	0.03
	*p*	0.144	0.694	0.798	0.666	0.674
Total cholesterol	r	0.103	0.004	0.033	0.084	0.077
	*p*	0.148	0.959	0.646	0.237	0.279
HbA1c	r	0.292	0.300	−0.197	0.008	−0.232
	*p*	<0.001	<0.001	0.005	0.907	0.001
HOMA-IR	r	0.212	0.287	−0.295	−0.024	−0.004
	*p*	0.003	<0.001	<0.001	0.739	0.959
Insulin	r	0.157	0.200	−0.209	0.024	−0.044
	*p*	0.026	0.004	0.003	0.740	0.533
Uric acid	r	0.028	0.153	0.092	0.075	−0.207
	*p*	0.698	0.031	0.195	0.293	0.003
CRP	r	0.193	−0.098	−0.026	0.063	−0.062
	*p*	0.006	0.167	0.714	0.378	0.38
Microalbuminuria	r	0.177	0.097	−0.144	−0.089	0.362
	*p*	0.012	0.171	0.042	0.211	<0.001

**Table 5:** Regression analysis of the occurrence of microvascular and macrovascular complications in patient group.

	Microvascular Complications			Macrovascular Complications		
Variable	OR	95% Cl (Lower–Upper)	*p*	OR	95% Cl (Lower–Upper)	*p*
Asprosin	1.082	1.048–1.116	<0.001	1.028	1.001–1.057	0.043
Clusterin	1.058	1.017–1.101	0.005	1.019	0.982–1.057	0.331
ZAG	0.392	0.974–1.043	0.186	17.068	0.874–0.955	0.001
NF-κB	1.508	1.046–2.174	0.028	1.261	0.892–1.804	0.204
PPAR-γ	0.896	0.840–0.955	0.001	0.998	0.943–1.056	0.946

## Data Availability

The original contributions presented in the study are included in the article, further inquiries can be directed to the corresponding author.
